# Optimization of Propidium Monoazide qPCR (Viability-qPCR) to Quantify the Killing by the *Gardnerella*-Specific Endolysin PM-477, Directly in Vaginal Samples from Women with Bacterial Vaginosis

**DOI:** 10.3390/antibiotics11010111

**Published:** 2022-01-15

**Authors:** Agnieszka Latka, Leen Van Simaey, Marijke Reynders, Piet Cools, Tess Rogier, Barbara Lebbe, Lorenzo Corsini, Christine Landlinger, Mario Vaneechoutte

**Affiliations:** 1 Laboratory Bacteriology Research, Department of Diagnostic Sciences, Faculty of Medicine and Health Sciences, Ghent University, 9000 Ghent, Belgium; Leen.VanSimaey@UGent.be (L.V.S.); Piet.Cools@UGent.be (P.C.); Tessrogier@outlook.com (T.R.); Mario.Vaneechoutte@UGent.be (M.V.); 2 Department of Pathogen Biology and Immunology, Institute of Genetics and Microbiology, University of Wroclaw, 51-148 Wroclaw, Poland; 3 Laboratory of Applied Biotechnology, Department of Biotechnology, Ghent University, 9000 Ghent, Belgium; 4 Laboratory Medicine, Medical Microbiology, AZ St.Jan Brugge-Oostende AV, 8000 Bruges, Belgium; Marijke.Reynders@azsintjan.be; 5 Department of Gynaecology, AZ St.Jan Brugge-Oostende AV, 8000 Bruges, Belgium; Barbara.Lebbe@azsintjan.be; 6 PhagoMed Biopharma GmbH, Vienna Biocenter, 1110 Wien, Austria; lorenzo.corsini@phagomed.com (L.C.); christine.landlinger@gmx.at (C.L.)

**Keywords:** endolysin, bacterial vaginosis, biofilm, *Gardnerella*, viability-qPCR

## Abstract

Quantification of the number of living cells in biofilm or after eradication treatments of biofilm, is problematic for different reasons. We assessed the performance of pre-treatment of DNA, planktonic cells and ex vivo vaginal biofilms of *Gardnerella* with propidium monoazide (PMAxx) to prevent qPCR-based amplification of DNA from killed cells (viability-qPCR). Standard PMAxx treatment did not completely inactivate free DNA and did not affect living cells. While culture indicated that killing of planktonic cells by heat or by endolysin was complete, viability-qPCR assessed only log reductions of 1.73 and 0.32, respectively. Therefore, we improved the standard protocol by comparing different (combinations of) parameters, such as concentration of PMAxx, and repetition, duration and incubation conditions of treatment. The optimized PMAxx treatment condition for further experiments consisted of three cycles, each of: 15 min incubation on ice with 50 µM PMAxx, followed by 15 min-long light exposure. This protocol was validated for use in vaginal samples from women with bacterial vaginosis. Up to log2.2 reduction of *Gardnerella* cells after treatment with PM-477 was documented, despite the complex composition of the samples, which might have hampered the activity of PM-477 as well as the quantification of low loads by viability-qPCR.

## 1. Introduction

Clinical microbiology is increasingly confronted with the consequences of biofilm formation, causing problems of chronic infections and chronic dysbiosis. This is also the case for bacterial vaginosis (BV), which often develops into a chronic and refractory condition and which is now generally recognized to be largely due to biofilm formation with *Gardnerella* as one of the principal bacterial taxa. Since antibiotic treatment only causes transient relief in many BV cases, alternative therapies such as endolysin treatment are increasingly being considered and developed. However, the effect of any therapy on a biofilm is difficult to assess because bacteria have already decreased metabolic activity in biofilms, which makes it challenging to evaluate therapy success. One option is qPCR, which has the disadvantage that by amplifying DNA from dead cells that is still present in the sample, it easily provides a false negative result (positive = therapy success). The second option, culturing, may provide false positive results if alive but dormant biofilm cells cannot be cultured under the same conditions as metabolically active planktonic cells.

In this study, we assessed to what extent viability-qPCR was a valuable tool to quantify biofilm eradication from vaginal samples by means of endolysin specific for *Gardnerella*. Viability-qPCR in principle is based on the ability of propidium monoazide (PMA) to block amplification of DNA outside of cells with intact cell membranes and of DNA inside of cells with damaged cell membranes, leaving only DNA from living cells amplifiable. In principle, PMA does not easily enter living cells, and therefore will bind only covalently with DNA from lysed cells, inhibiting amplification of DNA from lysed cells and enabling amplification of DNA from live cells only [1,2,3,4]. However, the method needs finetuning, since a PMA-treatment too weak will not inactivate all DNA from killed cells and PMA-treatment too harsh might also inactivate DNA inside living cells [5].

*Gardnerella* can be isolated from the urogenital tract of women and men [6,7,8] and can form biofilms on vaginal epithelial cells, observed as so-called clue cells in a condition known as BV [8,9], the most common reason of (malodorous) vaginal discharge. BV increases the risk of preterm delivery, human immunodeficiency virus infection and transmission as well as other sexually transmitted infections (STIs), intrauterine growth retardation, postpartum endometritis and pelvic inflammatory disease [10,11,12]. When growing as biofilm, *Gardnerella* changes gene expression, which may be one of the reasons of biofilm resistance towards antibiotics and the chronic nature of BV [9,13].

According to the Centers for Disease Control and Prevention, the antibiotics clindamycin or metronidazole are recommended to treat BV [14,15]. The level of their efficacy reaches only around 60%, with a high recurrence rate of 30–40% [14,16,17,18], which may also increase the dissemination of antibiotic resistance among non-*Gardnerella* BV-related species. Due to the lack of suitable and effective ways of treatment, there is the continuing need to find effective alternatives for antibiotics, with endolysins as a recent development e.g., [19,20]. Endolysins are bacteriophage-encoded enzymes that specifically degrade bacterial peptidoglycans resulting in a rapid bacteriolysis [19]. They are produced at the end of the phage replication cycle to lyse the bacterial host cell from within and enable phage progeny release [21]. Due to the lack of an outer membrane in the cell wall of Gram-positive bacteria, endolysins can be easily applied externally, leading to bacterial cell lysis. Moreover, endolysins were shown to be effective against bacterial biofilms and were characterized as antimicrobials against which the level of generated resistance is very low [20,22,23]. Together with their high killing specificity, leaving the commensal microflora unaffected, these characteristics make endolysins potential tools to fight antibiotic-resistant bacterial infections.

Here, we assessed the usefulness of PMA-based viability-qPCR to quantify killing by PM-477, a recently developed engineered endolysin, active against different *Gardnerella* species [20]. Therefore, we first optimized this approach for planktonic cells of *G. swidsinskii.* Finally, we quantified the efficacy of PM-477 on (dormant) *Gardnerella* cells in original biofilm as present in vaginal samples from women with BV.

## 2. Materials and Methods

### 2.1. Gardnerella Strains and Culture Conditions

*G. vaginalis* ATCC 14018^T^, *G. leopoldii* UGent 06.41^T^, *G. piotii* UGent 18.01^T^ and *G. swidsinskii* GS 10234^T^ were grown on chocolate (Choc) agar plates (Becton Dickinson, Franklin Lakes, NJ, USA) at 37 °C under anaerobic conditions (10% CO_2_, 10% H_2_, 80%N_2_, Concept 400, Anaerobic Workstation). Liquid cultures were prepared in New York City Broth III (10 mM HEPES (Sigma Aldrich, Burlington, MA, USA), 15 g/L Proteose Peptone No. 3 (Becton Dickinson), 3.8 g/L yeast extract (Thermo Fisher Scientific, Waltham, MA, USA), 86 mM sodium chloride (Carl Roth, Karlsruhe, Germany), 28 mM α-D-glucose (Sigma–Aldrich)), at pH 5 (NYB5), supplemented with 10% horse serum (HS, Thermo Fisher Scientific) (NYB5 + HS).

### 2.2. Preparation of PM-477 Phage Endolysin

Engineered phage endolysin PM-477 was described previously [20]. PM-477 was expressed and purified as presented previously [20]. Briefly, PM-477 expression in *E. coli* BL21 (DE3) was performed in Terrific Broth Medium, with induction with 1.5% α-lactose monohydrate (Carl Roth) at 25 °C for 24 h, with shaking at 250 rpm. Protein purification was performed by affinity chromatography on a nickel–nitrilotriacetic acid (Ni–NTA) HISTrap column. Elution with 50 mM MES (Carl Roth) pH 7, 150 mM NaCl (Carl Roth), 250 mM imidazole (Carl Roth) was followed by size exclusion chromatography and dialysis against MES buffer (50 mM MES (2-morpholinoethanesulfonic acid) pH 5.5, 100 mM NaCl, 8 mM MgSO_4_ (Sigma–Aldrich)). Protein concentration was determined at OD 260/280 nm or by using the PierceTM BCA (bicinchoninic acid) protein assay kit (Thermo Fisher Scientific). Purified PM-477 aliquots (0.5 mg/mL) of 450 µL were stored at −80 °C till the moment of use. The His tag used for purification was not cleaved off PM-477 for the scope of this study.

### 2.3. Inactivation of Extracted Gardnerella DNA Using PMAxx

Extracted *G. swidsinskii* GS 10234^T^ DNA at log8.00 genomes/mL was treated with PMAxx (PMA molecule optimized by Biotium, Fremont, CA, USA) according to the manufacturer’s protocol: 25 µM final concentration of PMAxx, 10 min incubation at room temperature, followed by 15 min light exposure in the PMA-Lite equipment (PMA-Lite™ LED Photolysis Device, for photoactivation of PMAxx™, PMA or EMA (Biotium), the PMA-Lite equipment illuminates each tube with 3 LEDs, one at the bottom and two at the side, of each 600–800 milliCandela (0.6–0.8 lumen) at a wavelength of 465–475 nm). Finally, DNA PMAxx-treated and non-treated were used for qPCR specific for *G. swidsinskii.*

### 2.4. Viability-qPCR on Live and Heat Killed Gardnerella Cells

Cells of *G. swidsinski* GS 10234^T^, freshly cultured on Choc agar plates, were used to prepare a bacterial suspension at OD_600_ = 0.1 in NYB5 + HS, corresponding to log8.3 CFU/mL. Suspension was divided into “live” and “dead” samples. “Dead” samples were heat killed by incubation at 95 °C for 10 min. Aliquots were centrifuged for 10 min at 4000× *g* in a benchtop centrifuge (Biofuge Pico, Heraeus, Sysmex, Hoeilaart, Belgium) and the supernatant was removed. Standard PMAxx treatment conditions was performed (25 µM final concentration of PMAxx, 10 min incubation at room temperature, followed by 15 min light exposure in the PMA-Lite equipment) and cells were pelleted (10 min 5000× *g*). Finally, DNA was extracted and used for qPCR specific for *G. swidsinskii.*

### 2.5. Optimization of Viability-qPCR

Viability-qPCR was optimized using *G. swidsinski* GS 10234^T^. Cells of *G. swidsinski* GS 10234^T^, freshly cultured on Choc agar plates, were used to prepare a bacterial suspension at OD_600_ = 0.1 in NYB5 + HS, corresponding to log8.3 CFU/mL. Then *G. swidsinkii* cells were treated with 0.05 mg/mL PM-477 overnight at 37 °C in anaerobic conditions (Concept 400, Anaerobic Workstation, Ruskinn, Bridgend, United Kingdom, 75% humidity). As a control, treatment with MES buffer was performed. After overnight treatment, aliquots were centrifuged for 10 min at 4000× *g* in a benchtop centrifuge (Biofuge Pico, Heraeus) and the supernatant was removed. Different PMAxx treatment conditions and addition of the apolar solvent dimethylsulfoxide (DMSO 2%-which might increase permeability of the cell membranes of killed cells and is used as the solvent for the alternative dye, ethidium monoazide bromide (EMA) [1]) were tested on pelleted cells, as indicated in Table 1. During PMAxx treatment, the samples were protected from light. After PMAxx treatment, vials were exposed to high intensity light for 15 min in the (PMA-Lite equipment and pelleted (10 min 5000× *g*). Finally, DNA was extracted, and the DNA extracts were used for qPCR specific for *G. swidsinskii.*

As an additional control to assess the bactericidal effect of PM-477, each endolysin-treated sample was diluted in 10-fold dilution series, plated on Choc agar plates and cultured. After 48 h of incubation at 37 °C in anaerobic conditions, colonies were counted and CFU/mL was calculated.

### 2.6. Optimized Viability-qPCR Protocol (PMAxx Treatment Followed by qPCR) for Differentiation between Live and Dead Cells

Two aliquots of vaginal samples were incubated with MES and two with PM-477 endolysin, and all four were treated with 50 µM PMAxx for 15 min on ice protected from light followed by 15 min light exposure in the PMA-Lite equipment. This procedure was repeated three times. Subsequently, the aliquots were centrifuged for 10 min at 5000× *g* in a benchtop centrifuge and supernatant was removed. Pellets were used for DNA extraction and qPCRs, as described below.

### 2.7. Influence of Dead Cells on the Quantification of Live Cells

Cells of *G. swidsinskii* GS 10234^T^, freshly cultured on Choc agar plates, were used to prepare a bacterial suspension at OD_600_ = 1 and OD_600_ = 0.1 in NYB5 + HS, corresponding to log9.3 and log8.3 CFU/mL respectively. Part of the cell suspensions was heat killed by incubation at 95 °C for 10 min. Ten-fold dilution series of living cells were prepared in heat killed suspensions to obtain variable titers of living cells from log9.3 to log3.3 in a constant titer of log9 killed cells and from log8.3 to log3.3 of living cells in a constant titer of log8 killed cells. Aliquots were centrifuged for 10 min at 4000× *g* in a benchtop centrifuge (Biofuge Pico, Heraeus) and the supernatant was removed. Optimized PMAxx treatment protocol was performed (50 µM final concentration of PMAxx, 15 min incubation at room temperature, followed by 15 min light exposure in the PMA-Lite equipment) and cells were pelleted (10 min 5000× *g*). Finally, DNA was extracted, and the DNA extracts were used for qPCR specific for *G. swidsinskii.*

### 2.8. Validation of Viability-qPCR on Planktonic Cells of Different Gardnerella Species

Freshly cultured cells of Gv17 (*G. piotii* UGent 18.01^T^), Gv10 (*G. leopoldii* UGent 06.41^T^), Gv9 (*G. vaginalis* ATCC 14018^T^) and Gv23 (*G. swidsinskii* GS 10234^T^) were used to prepare aliquots at OD_600_ = 0.1 in NYB5 + HS. Then *Gardnerella* cells were treated with 0.05 mg/mL PM-477 overnight at 37 °C in anaerobic conditions at 75% humidity (Concept 400, Anaerobic Workstation). As a control, treatment with MES buffer was performed. After overnight treatment, aliquots were centrifuged for 10 min at 4000× *g* in a benchtop centrifuge and supernatant was removed. PMAxx treatment was performed on the pelleted cells according to the PMAxx treatment protocol that had been optimized (as described above), and followed by DNA extraction and species-specific qPCRs.

### 2.9. Vaginal Swabs Collection

Vaginal samples were collected from randomly selected women who were previously diagnosed with BV. Fresh vaginal smears were collected with ESwab™ (COPAN, Murrieta, CA, USA) and resuspended in liquid Amies. The vials were frozen in −80 °C and stored until further use.

### 2.10. Differentiation between Live and Dead Gardnerella after PM-477 Treatment in Vaginal Samples from Women with Bacterial Vaginosis

Each vaginal sample was vortexed and divided into five equal aliquots of 45 µL. Respectively three aliquots were treated with MES buffer (50 mM MES, 100 mM NaCl, 8 mM MgSO_4_, pH 5.5) and two were treated with PM-477 (final conc. 0.1 mg/mL in MES) overnight at 37 °C in anaerobic conditions at 75% humidity (Concept 400, Anaerobic Workstation). After overnight treatment, the aliquots were centrifuged for 10 min at 4000× *g* in a benchtop centrifuge and the supernatant was removed. PMAxx treatment was performed on the pelleted cells of 2 MES treated and 2 PM-477-treated samples according to the optimized conditions (as described above). One aliquot was treated with MES buffer only and remained without PMAxx incubation. Thereafter, DNA extraction and species-specific qPCRs were carried out.

### 2.11. DNA Extraction of Gardnerella Planktonic Cells and Vaginal Samples after PM-477 Treatment Followed by PMAxx Treatment

Samples, after PM-477 treatment followed by PMAxx treatment, were centrifuged for 10 min at 5000× *g* in a benchtop microcentrifuge (Biofuge Pico, Heraeus), and the supernatant was removed. Pellets were protease digested by adding 400 µL of protease buffer (9.5 mL of a 20 mM Tris-HCl pH 8.0, 0.5 mL of 10% SDS) containing 0.6 mg/mL Proteinase K (Merck, Darmstadt, Germany) per sample. Thereafter, samples were pre-lysed with Nuclisens EasyMag Lysis Buffer (bioMerieux, Marcy-l’Étoile, France) and frozen at −80 °C. Finally, DNA was extracted with the High Pure PCR Template Preparation Kit (Roche, Basel, Switzerland) and stored at −20 °C until further use. On the DNA extracts, *Gardnerella* species-specific or *Lactobacillus* genus-specific qPCRs were performed.

### 2.12. Design of Gardnerella Species-Specific Primers

After a literature search, six different household genes (i.e., *Xfp*, *clpC*, *dnaJ*, *priA*, *rpoB* and *dnaG*) were considered for the design of *Gardnerella* species-specific primers. The annotation tool, DFAST, was used to find the sequences of these genes. The sequences from the different *Gardnerella* species were aligned using Clustal Omega, with most variation found for *dnaG*. Therefore, *Gardnerella* species-specific primers (Table 2) targeting the DnaG region were designed using SnapGene and ordered at Eurogentec.

### 2.13. Quantification of G. leopoldii, G. piotii, G. swidsinskii, G. vaginalis and the Genus Lactobacillus by Means of qPCR

*Gardnerella* species-specific qPCRs were performed using SYBR Green qPCR Master Mix (Roche) and species-specific primers (500 nM final concentration,) with the following cyclic conditions: 95 °C denaturation for 5 min, followed by 40 amplification cycles of 15 s denaturation at 95 °C, 30 s annealing at 56 °C and 30 s extension at 72 °C, followed by melting analysis between 55 and 95 °C with a ramp rate of 2.5 °C/s.

*Lactobacillus* genus-specific qPCR were performed using LightCycler^®^ 480 High Resolution Melting Master (Roche) and genus specific primers (300 nM final concentration, Table 2) and with the following cyclic conditions: initial denaturation by heating to 95 °C during 10 min, followed by 45 cycles of 15 sec at 95 °C, 40 sec at 58 °C and 30 sec at 72 °C, followed by melting analysis between 55 and 95 °C with a ramp rate of 2.5 °C/s.

In each qPCR assay, a 10-fold standard dilution series of genomic DNA was run in duplicate, to calculate the concentrations of the unknown samples. All concentrations were expressed in genome equivalents per ml (genomes/mL).

The obtained Cq-values (quantification cycle) of these qPCR assays were expressed as bacterial concentrations (genomes/mL) by calculations of the software based on a standard curve (genomes/mL = 10^(Cq–intercept)/slope)^).

Values of efficiency, slope and intercept were as follows: 1.864, −3.698, 49.20 for *G. leopoldii* 1.785, −3.973, 52.57 for *G. piotii*, 1.822, −3.839, 51.97 for *G. swidsinskii*, 1.910, −3.559, 47.38 for *G. vaginalis* and 1.903, −3579, 46.76 for *Lactobacillus*, respectively.

### 2.14. Statistical Analysis

One-way ANOVA followed by Tukey’s multiple comparisons test (GraphPad Prism 9, San Diego, CA, USA) were run to test if there was a statistically significant difference in log(genomes/mL) reduction between samples treated and not treated with PM-477 followed by PMAxx as well as to test if PM-477 and PMaxx treatment had a significant effect in log(genomes/mL) reduction of different *Gardnerella* strains. Normality and lognormality (Anderson–Darling test, D’Agostino & Pearson test, Shapiro–Wilk test and Kolmogorov–Smirnov test) test followed by Wilcoxon test were run to compare if there was a statistically significant difference in log(genomes/mL) between vaginal samples treated and not treated with PM-477. Spearman’s rank correlation coefficient was calculated to assess the correlation between initial load of *Gardnerella* and reduction caused by PM-477 treatment. *p*-values of < 0.05 were considered statistically significant, and this was indicated with asterisks in the graphs.

## 3. Results

In order to quantify the effect of the endolysin PM-477 on the lysis of *Gardnerella* cells in BV samples, we first optimized the viability-qPCR approach.

### 3.1. Optimization of Viability-qPCR

To set up the method, multiple parameters of PMAxx treatment were tested on planktonic *Gardnerella swidsinskii* cells (live, heat-killed and PM-477-killed) and on DNA extracted from these cells. First, we treated purified *G. swidsinskii* DNA at log8.90 genomes/mL with PMAxx according to the manufacturer’s protocol, i.e., 25 µM final concentration of PMAxx, 10 min incubation at room temperature, followed by 15 min light exposure in the PMA-Lite equipment. This resulted in a reduction of log4.35 genomes/mL (±0.00), with reaching the limit of detection (Cq = 35). As such, standard PMAxx treatment was able to inactivate free DNA to below the limit of detection of the qPCR (i.e., < log4.55 genomes/mL) (Table S1).

Subsequently, we tested the ability of PMAxx to differentiate between live and heat-killed (10 min 95 °C) planktonic cells of *G. swidsinskii*. When heat-killed planktonic *Gardnerella* cells at starting OD_600_ = 0.1 (log8.19 (±0.17) CFU/mL) were treated with PMAxx, using the conditions recommended by the manufacturer, the observed log reduction in viability-qPCR was log1.73 (±0.05) (Table S2). To assess the level of PMAxx-induced reduction of living cells, we also compared live cells not treated with PMAxx to live cells treated with PMAxx, and found no significant difference (log0.05 ± log0.1).

When *Gardnerella* cells, that had been killed by overnight anaerobic incubation with 0.05 mg/mL of PM-477 at 37 °C and 75% humidity, were subjected to the standard PMAxx treatment protocol as recommended by the producer, no statistically significant log reduction, i.e., only log0.32 (±0.13), was observed. However, culture confirmed that heat as well as the PM-477 treatment led to (at least) log6.19 reduction in CFU/mL (reaching the limit of detection of the culture assay, i.e., log2.00 CFU/mL) (Table S3).

**Figure 1:**
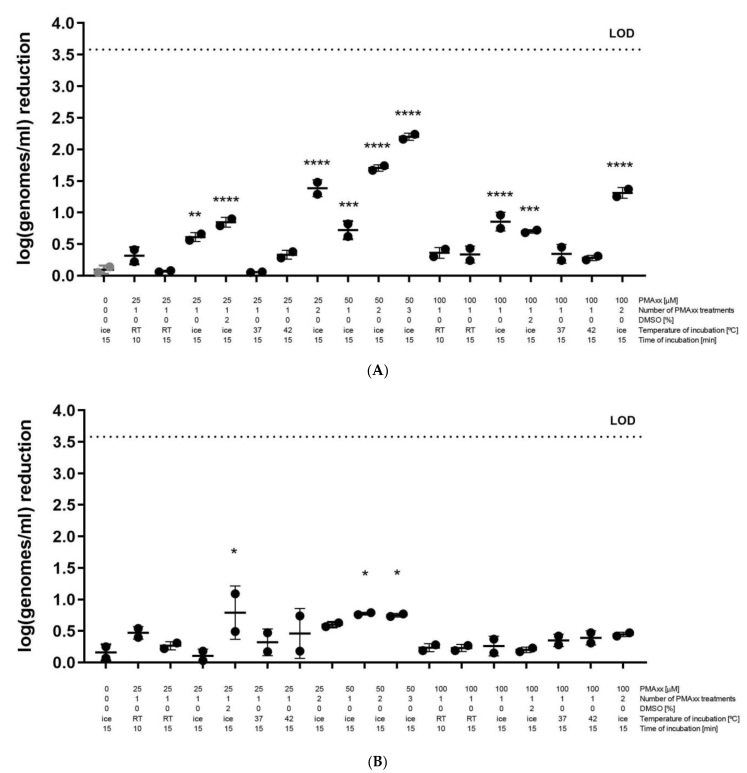
(**A**) Reduction of cells (log genomes/mL), killed by 0.05 mg/mL PM-477 during overnight anaerobic incubation at 37 °C in 75% humidity, as assessed by different PMAxx treatment conditions of the killed cells, followed by DNA extraction and *Gardnerella swidsinskii* specific qPCR. Values are calculated as: log(number of MES-treated PMAxx-treated genomes/number of PM-477-killed PMAxx-treated genomes). (**B**) Reduction of cells (log genomes/mL), not treated with PM-477, i.e., reduction due to PMAxx treatment itself. Values are calculated as: log(number of MES-treated PMAxx-non-treated genomes/number of MES-treated PMAxx-treated genomes). Grey dots represent samples on which PMAxx treatment was not performed, black dots represent samples on which different PMAxx treatments were performed. *: *p*-value  <  0.05; **: *p*-value  < 0.01; ***: *p*-value  <  0.001; ****: *p*-value  <  0.0001, as assessed by one-way ANOVA followed by Tukey’s multiple comparisons test in GraphPad Prism 9. Asterisks indicate statistically significant difference between samples treated with PM-477 and inactivated by PMAxx treatment (black dots) and samples treated with PM-477 only (grey dots, 1A). B. Asterisks indicate statistically significant difference between samples treated with MES and inactivated by PMAxx treatment and samples treated with MES only (assumed to be 0).

Because of the disagreement between culture and viability-qPCR for PM-477- and heat-killed cells, different PMAxx treatment conditions were tested in order to maximize the reduction observed for PM-477-killed cells by means of viability-qPCR. PM-477-killed cells and live control cells log8.19 (±0.17) cells, incubated with PM-477 in MES buffer or with MES buffer alone, were subjected to combinations of three different final PMAxx concentrations (25 µM, 100 µM and 50 µM), incubation with PMAxx at four different temperatures (on ice, at room temperature, at 37 °C and at 42 °C), and addition of the dimethylsulfoxide (DMSO 2%)—(Table 1, Figure 1). None of the abovementioned conditions reached log6.19 reduction, with the average log reduction ranging from log0.06 (±0.01) for incubation at 37 °C with 25 µM PMAxx to log0.84 (±0.08) for on ice incubation with 25 µM PMAxx and 2% DMSO and log0.86 (±0.15) for on ice incubation with 100 µM PMAxx. However, there was stronger, statistically significant reduction only when PMAxx incubation was performed on ice, instead of at room temperature or at 37 °C or at 42 °C. Therefore, and also because incubation on ice is a more reproducible condition than incubation at room temperature, in the next step, we compared the influence of two or three repetitive PMAxx treatments, all on ice.

There was a tendency to stronger reduction when repeating PMAxx treatment twice on ice, with reductions of log1.31 (±0.09) and log1.38 (±0.13) for treatment with 100 µM and 25 µM PMAxx, respectively. The highest statistically significant reduction was observed for 50 µM PMAxx, repeated three times, i.e., log2.20 (±0.06) reduction, *p*-value < 0.0001 (Figure 1A). The log reduction of viability-qPCR for the live cells in MES buffer was relatively high for repetitive PMAxx treatments (log0.75 ± 0.03), compared to the reduction caused after single PMAxx treatment (below log0.5, except for the treatment performed with 25 µM PMAxx at 37 °C (log0.79 ± 0.43) and with 50 µM PMAxx on ice (log0.60 ± 0.04) (Figure 1B).

Apart from the conditions summarized in Figure 1, we further also modified the PM-477 treatment conditions: incubation in high (75%) versus low (60%) humidity and 5 h long treatment versus overnight treatment. All four variables resulted in the same strong reduction of living cells when assessed by means of culture (log6.19) but did not improve the reduction as assessed by viability-qPCR (data not shown).

Supplementation of three NaCl concentrations (0, 200 mM and 400 mM) and the addition of different concentrations of dimethyl sulfoxide (DMSO 0%, 1%, 2% and 5%,), 1 mM ethylenediamine tetraacetic acid (EDTA, also shown to increase membrane permeability [24]), and sublethal concentrations (0.01%, 0.002%, 0.0001%) of sodium dodecyl sulphate (SDS, used for cell lysis in RNA and DNA extraction procedures) along with a longer pre-incubation time (30 min), were also tested (data not shown). We could not observe statistically significant improvement of PMAxx-induced inhibition of DNA-amplification from PM-477 killed cells.

Based on the above results, whereby triple PMAxx treatment with 50 µM showed the highest reduction for the PM-477-killed cells, the standard PMAxx treatment condition for further experiments was chosen as three cycles of 15 min incubation on ice with 50 µM PMAxx, followed by 15 min-long light exposure.

To test the influence of dead cells on the quantification of live cells, we prepared mixtures of variable amounts of log4 to log9 living cells with log8 or log9 of dead cells (heat-killed, 95 °C, 10 min). For *Gardnerella* live/dead mixtures, we observed that dead to live cells ratios exceeding 100:1 caused overestimation of the number of live cells (Table S4). PMAxx-qPCR of heat killed cells did not reach the limit of detection and reached a maximum of log2.58 and log2.68 reductions for dead cells at log9 and log8 respectively.

### 3.2. Influence of PMAxx Treatment on Different Gardnerella Species

**Figure 2:**
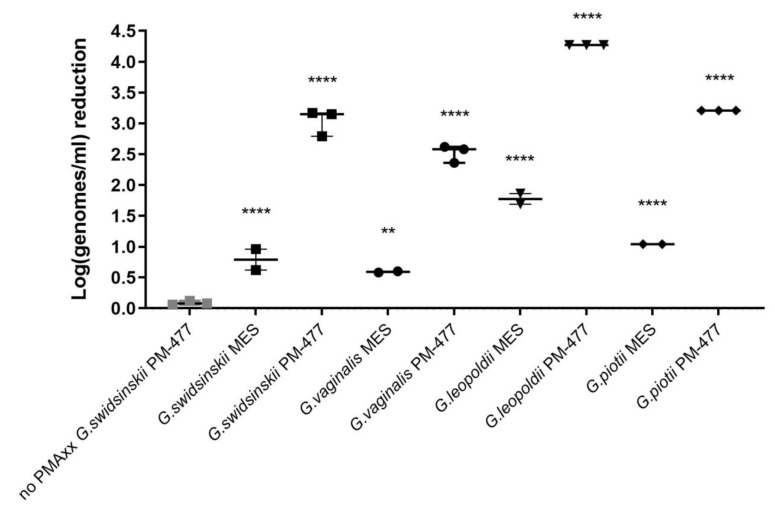
Reduction of cells (log(genomes/mL)), killed by 0.05 mg/mL PM-477 during overnight anaerobic incubation at 37 °C in 75% humidity, as assessed by optimized PMAxx treatment conditions (15 min on ice incubation with 0.05 mM PMAxx, followed by 15 min light exposure, repeated three times), DNA extraction and species-specific qPCR. Values are calculated as: log(number of MES-treated PMAxx-treated genomes/number of PM-477-killed PMAxx-treated genomes) and reduction of cells (log(genomes/mL)), not treated with PM-477, i.e., reduction due to PMAxx treatment itself. Values are calculated as: log(number of MES-treated PMAxx-non-treated genomes/number of MES-treated PMAxx-treated genomes). Legend: triangle: *G. leopoldii*, rhombus: *G. piotii*, square: *G. swidsinskii*, circle: *G. vaginalis,* grey symbols correspond to samples not treated with PMAxx, black symbols correspond to samples treated with PMAxx.; **: *p*-value < 0.01;; ****: *p*-value  <  0.0001, as assessed by one-way ANOVA followed by Tukey’s multiple comparisons test in GraphPad Prism 9. Asterisks indicate statistically significant difference: between samples treated with MES for which DNA was inactivated with PMAxx and samples treated with MES only (reduction of which was assumed to be 0 for each species); between samples treated with PM-477 followed by DNA inactivation with PMAxx compared to MES-treated PMAxx-treated samples of respective species. Only for *G. swidsinskii* were there samples treated with PM-477 but not followed by DNA inactivation with PMAxx and no statistically significant difference could be observed compared to MES noPMAxx (reduction of which was assumed to be 0). No reduction is expected for samples for which the DNA is not inactivated by PMAxx treatment.

Before proceeding with vaginal samples, we tested the influence of the optimized PMAxx procedure on cultured cells of the type strains of the four named *Gardnerella* species. Figure 2 shows that the optimized PMAxx treatment also causes reduction for live cells, tested at starting concentrations at log9.00 (except for *G. swidsinskii* at log8.89), ranging between log0.59 ± 0.01 (*G. vaginalis*) and log1.77 ± 0.12 (*G. leopoldii*). Still, a statistically significant reduction could be observed for endolysin-killed cells of all species. Observed reduction of PM-477-killed cells was log3.04 (±0.22) for *G. swidsinskii*, log2.52 (±0.14) for *G. vaginalis,* log4.27 (±0.00) for *G. leopoldii* and log3.21 (± 0.00) for *G. piotii*, with reduction of live cells being log0.79 (±0.24), log0.59 (±0.01), log1.77 (±0.12) and log1.04 (±0.00), respectively.

### 3.3. Assessing the Bactericidal Efficacy of PM-477 on Gardnerella in Vaginal Samples from Women with Bacterial Vaginosis, by Means of Optimized Viability-qPCR

**Figure 3:**
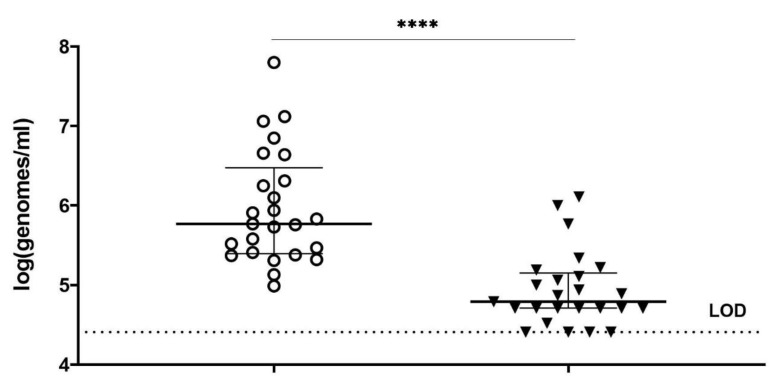
Reduction of cells log(genomes/mL) obtained with *Gardnerella* species-specific qPCRs for vaginal samples from women with BV, untreated (only MES buffer, transparent circles) and treated with 100 µg/mL PM-477 (black triangles) overnight. ****: *p*-value  <  0.0001, as assessed by Wilcoxon test. LOD—limit of detection.

Finally, the optimized viability-qPCR was tested on ex vivo BV samples. Subsequently, the optimized PMAxx procedure was applied to quantify the effect of 100 µg/mL of PM-477 (twice higher than used for planktonic cells) (overnight anaerobic incubation, 37 °C, 75% humidity) onto the killing of *Gardnerella* in vaginal samples from women with BV. In summary, the Cq-values obtained for four *Gardnerella*-specific qPCRs after treatment of the vaginal samples with PM-477 were close to or reached the limit of detection (Cq value 35) for 12 out of 25 samples (Figure 3). The difference between samples treated with only MES and samples treated with PM-477 was statistically significant (*p* < 0.0001). *G. vaginalis* was the species most frequently detected, i.e., in 13 samples. The sample with the lowest quantity of live cells (log5.1) showed the lowest reduction (log0.4) and the sample with the highest quantity of live cells (log7.8) showed a reduction of log1.8. The highest reduction (log2.2) was observed for a sample with log6.6 live cells. *G. swidsinskii* was detected in three samples, ranging between log5.4 and log6.6 of live cells and the corresponding level of reduction ranged between log0.9 and log2.2. Eight samples were positive for *G. leopoldii*, with number of live cells ranging between log5 and log6.7, and reduction ranging between log0.3 and log1.4. One sample was found to be positive for *G. piotii,* with log5.8 live cells and log1.4 reduction.

**Figure 4:**
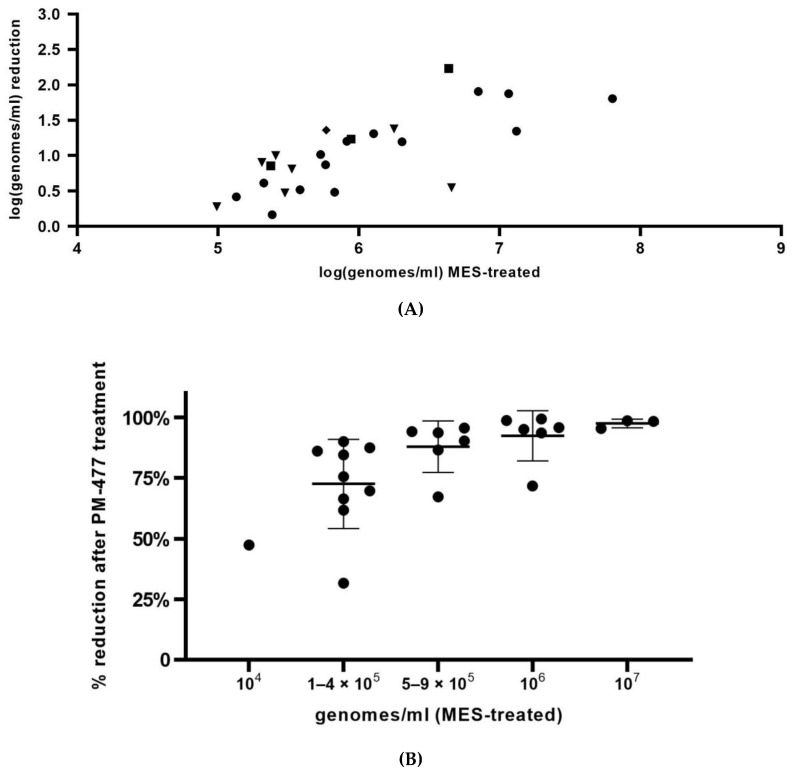
(**A**) Reduction of number of *Gardnerella* genomes by overnight treatment of vaginal samples with 100 µg/mL PM-477, as assessed by Gardnerella species-specific qPCRs, calculated as: log(MES/PM-477). (**B**) Percentage of reduction of Gardnerella genomes by overnight treatment of vaginal samples with 100 µg/mL PM-477, as assessed by Gardnerella species-specific qPCRs, calculated as: (MES minus PM-477)/MES. MES = number of genomes after treatment of cells with MES and inactivation of free DNA with PMAxx and PM-477 = number of genomes after treatment of cells with PM-477 and inactivation of free DNA with PMAxx. Legend: triangle: *G. leopoldii*, rhombus: *G. piotii*, square: *G. swidsinskii*, circle: *G. vaginalis*.

Most of the samples had an initial low load of living cells of *Gardnerella* (high Cq values, close to the limit of detection), making it difficult to document further reduction by PM-477 treatment. However, we could show that, the higher the initial value of log genomes/mL (low Cq values), the stronger the reduction by PM-477 treatment (Figure 4A, r = 0.9823). We determined the percentage reduction of *Gardnerella* species that was documented for each sample by means of viability-qPCR. Data are shown in Figure 4B, whereby samples have been categorized according to starting load of *Gardnerella* cells. We obtained reductions of more than 75% for most samples, with the strongest reductions, up to 98.7%, for the samples with the highest initial loads (log7 genomes/mL, as determined after MES treatment).

**Figure 5:**
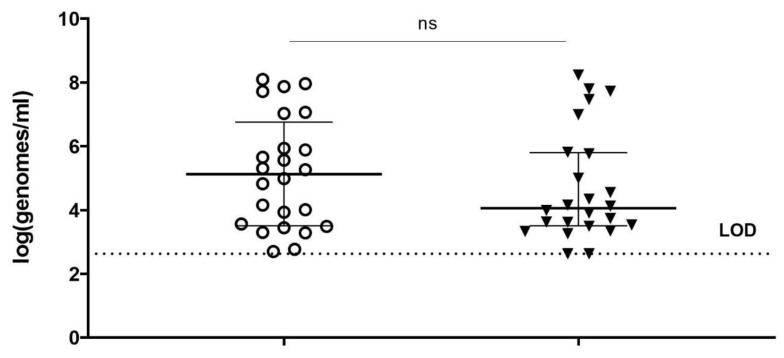
Reduction of cells log(genomes/mL) obtained with PMAxx and genus *Lactobacillus* genus-specific qPCRs for vaginal samples from women with BV, untreated (transparent circles) and treated with 100 µg/mL PM-477 (black triangles) overnight, shown for *Lactobacillus* and determined with PMAxx-genus specific qPCR Non significant (ns) as assessed by Wilcoxon test. LOD—limit of detection, ns—non significant.

Different *Lactobacillus* species are present in vaginal econiche and are crucial to keep a low pH and protect against vaginal dysbiosis. We wanted to check if PM-477 will not harm the commensal vaginal microflora. Therefore, apart from performing *Gardnerella* species-specific qPCRs, we also performed *Lactobacillus* genus specific viability-PCR. The same BV samples as analyzed for *Gardnerella* did not indicate statistically significant reduction of the number of *Lactobacillus* cells (Figure 5, *p* = 0.0726). Among these samples, high variability in *Lactobacillus* loads was observed, as could be expected.

## 4. Discussion

The goals of this study were: (1) to determine the optimal conditions of viability-qPCR to specifically differentiate between live and dead *Gardnerella*/*Lactobacillus* cells in vitro and in vaginal samples of women with bacterial vaginosis (BV), and (2) to quantify the effect of *Gardnerella*-specific endolysin PM-477 in vaginal samples of women with BV.

The in vitro efficacy of PM-477 (tested in this study) to lyse *Gardnerella* cells was recently shown [20] on a broad panel of *Gardnerella* strains belonging to four *Gardnerella* species (*G. leopoldii*, *G. piotii*, *G. vaginalis* and *G. swidsinskii*), known to be important in vaginal microbiology [25]. At the same time, PM-477 is inactive against *Lactobacillus* species [20] typically present in the vagina [26]. Quantification of the number of viable cells of vaginal bacterial taxa such as *Gardnerella* in vaginal samples is cumbersome because of (i) the polymicrobial vaginal environment, (ii) the difficulty to selectively culture *Gardnerella* strains and (iii) the likeliness that culture is false-negative due to dormancy of the cells in vaginal biofilms [8,9]. As such, culture could lead to false-positive results when trying to assess the in vitro antimicrobial effectiveness of agents such as PM-477, an engineered endolysin specifically active against *Gardnerella* species [20]. However, DNA and RNA qPCR methods are also problematic when it comes to quantifying the number of viable cells. DNA qPCR may lead to false-negative results, since also DNA from cells that were efficiently killed will be amplified. RNA qPCR might lead to false-positive results since living cells in biofilms may reduce metabolism and consequently also may reduce DNA transcription into RNA. Since differentiation between viable versus killed cells is crucial to quantify the bactericidal efficacy of potential antimicrobials, we decided to optimize a viability-qPCR method, which consisted of a combination of propidium monoazide (PMAxx, molecule optimized by Biotium) prior treatment, DNA extraction and *Gardnerella* species-specific qPCRs.

Viability-qPCR assesses cell viability on the basis of the impermeability of the cell envelope of living cells for propidium monoazide [1,27,28]. Propidium monoazide can bind to unprotected DNA molecules (from nonviable cells with impaired membranes and with free nucleic acids from lysed cell) by means of photoactivation, such that these nucleic acids can no longer serve as template for the polymerase chain reaction. Viable cells, on the other hand, prevent the contact of PMA with their genomic DNA, which can further serve as the matrix for amplification. In principle, only DNA from viable cells is amplified and quantified.

In practice, at least some DNA from dead cells escapes PMA binding, for different reasons, such as adsorption onto debris of degraded cells or due to insufficient cell lysis of killed cells, and this results in overestimation of the number of living cells by means of viability-qPCR. Conversely, some DNA from viable cells may become covalently bound to PMA, for different reasons, such as penetration of (excess) PMA into intact cells or killing and lysis of live cells by the treatment with PMA. This might result in an underestimation of the number of living cells by means of viability-qPCR. The importance of the contribution of these factors can differ between target organisms, bactericidal/bacteriolytic agents, sample types as well as DNA binding agents [29,30].

Therefore, the reliability of this approach relies on an appropriate trade-off between sufficiently harsh conditions to ensure that all DNA from dead cells is bound to the PMA dye, but at the same time, conditions that avoid damage and subsequent PMA-labelling of DNA from cells that were alive prior to the PMA treatment. Moreover, the reliability of viability-qPCR also depends on the correlation between the death of a bacterial cell and the increase of cell membrane impermeability. It is conceivable that no longer viable cells can still have intact membranes. Cell death without cell lysis or cell death not resulting in sufficient cell permeability of the cytoplasmic membrane will cause overestimation of the number of living cells. Therefore, the effect of bacteriostatic and bactericidal agents cannot be quantified by means of viability-qPCR. In contrast, killing by bacteriolytic agents, such as endolysins, should be quantifiable in a reliable manner.

Because of all the above-mentioned possible constraints and biases of viability-qPCR, we compared different parameters of the application of PMA to inhibit as completely as possible the amplification of DNA from dead cells without jeopardizing the integrity of the membranes of non-killed cells. This was carried out to quantify the ex vivo efficacy of PM-477, a genetically-engineered endolysin recently described [20] and aimed to be a therapy for BV, that may be more efficient than current antibiotic-based treatments.

First, we opted to use PMA instead of EMA, because PMA has been shown to be more efficiently excluded from cells with intact cell membranes [1], possibly due to its higher charge relative to EMA [1]. PMA is related to propidium iodide, used for life/dead staining, but with an azide group instead of an amino group on the phenanthridine ring, which enables photo-induced cross-linking to dsDNA such as removing the chemically modified DNA as a template for qPCR.

Dye permeability of lipid bilayers depends to a large extent on temperature, whereby elevated temperatures during sample treatment have been shown to increase dye uptake. One study found that for *Salmonella typhimurium* and *Listeria monocytogenes*, PMA-based inhibition of amplification of DNA from dead cells was improved at temperatures up to 40 °C, while signals from live cells were not (*Salmonella*) or only slightly affected (*Listeria*) [4]. Therefore, we compared PMAxx incubation on ice, at RT, 37 °C and 42 °C. We observed significantly better PMAxx penetration into the dead cells for incubation on ice, compared to incubation at RT, 37 and 42 °C.

Pan and Breidt [2] reported no penetration of PMA to live *L. monocytogenes* cells. For single PMAxx treatment, we found a log0.60 (±0.04) signal decrease of viable *G. swidsinskii* and for triple treatment, we observed a log0.6–log1.65 decrease of signal for four *Gardnerella* species, which were statistically significant (*p* value < 0.05).

Kralik et al. [31] compared single, double and triple PMA treatment (incubation during 5 min at RT) of around log8 CFU/mL cultured cells of a *Mycobacterium avium* isolate and found that in case of double PMA treatment, the signal decrease of dead cells was about 1.5 cycles higher than in case of single treatment, thus increasing sensitivity. However, triple PMA treatment resulted in signal decrease for both live and dead cells. Although *Gardnerella* has a thin Gram-positive cell wall [32], whereas *M. avium* is characterized by a thick cell wall, whereby the presence of mycolic acids may further decrease PMA penetration [33]. To understand how many cycles of PMA treatment may be optimal for *Gardnerella*, we also compared single, double and triple PMA treatments. In our study, double and triple treatment of live *Gardnerella* cells with 0.05 mM PMAxx resulted in the same level of signal decrease (log0.78 ± 0.02 and log0.75 ± 0.03, respectively). Signal reduction for PM-477 killed cells was log0.5 higher in case of three times PMAxx treatment compared to treatment performed two times (log1.7 ± 0.05 and log2.2 ± 0.06, respectively), which was statistically significant.

In the study of Pan and Breidt [2], double 0.05 mM PMA treatment of approx. 2.4 log7 *L. monocytogenes* cells at room temperature was considered as optimal, causing 2.8 cycles reduction compared to single PMA treatment. No further reduction was obtained with 3 × PMA treatment. The same authors reported that neither increased dye concentrations (0.10 and 0.20 mM) nor increased incubation temperatures (23 °C and 40 °C) showed significant additional reduction. In the present study, the repetitive treatment was also the most efficient, with the highest log reduction observed for three times PMA. Moreover, in agreement with Pan and Breidt [2], higher temperatures did not result in statistically significant stronger reduction than on ice incubation.

Viability-qPCR has been widely used, although the above literature review suggests that it is difficult to standardize and that treatment conditions (concentration of the dye, incubation time and temperature, number of treatments) should be adapted to each organism (and each sample type) studied. The efficacy of PMA-covalent binding as a means to inhibit PCR-based amplification of target DNA can be further jeopardized in case bacterial cells grow within clusters or biofilms. Pisz et al. [34] suggested that the composition of the biofilms, particularly their extracellular polymeric substances, may interfere with either the DNA binding or the photoactivation of EMA. This interference may be similar to the way biofilm matrix polymers protect matrix-bound extracellular DNA from environmental nucleases [35]. Furthermore, the extracellular biofilm DNA may reduce the concentrations of unbound PMA by binding it.

Another problem may be caused by the fact that the dye could adsorb (differently) onto different compounds present in the sample [36]. EMA/PMA efficacy can be complicated by the composition of natural samples, as was found even for samples such as environmental water [37], and which can be expected to be the case for clinical samples. Moreover, even samples of the same type, but from different individuals, may differ substantially with regard to their interaction with the DNA-binding compounds, as can be the case for vaginal swabs from women with BV. That might be one of the reasons why in our study, viability-PCR reached the detection limit for only half of the samples treated with PM-477. Viability-qPCR may be hampered as well by the presence of dead cells and by biofilms.

The presence of substantial amounts of dead cells also could interfere with DNA extraction or with amplification from viable cells. For example, the number of viable cells of *Escherichia coli* was underestimated by viability-qPCR, when log8 dead and live cells per ml were present [38]. We observed that when the ratio of dead to live cells exceeded 100:1, the number of live cells was overestimated. For *Listeria monocytogenes*, Pan and Breidt [2] reported that the ratio of dead cells to the live cells could be no greater than log5. Our results are consistent with those from Yang et al. [3], who showed that a linear relationship between the log numbers and Cq value of live cells can be achieved only when viable cells comprise between 1 and 100% of the mixture of live and dead bacteria. When live bacteria were below 0.1% of the mixture, an increase in Cq value was not corresponding to the decrease in live cells. Similarly, Lovdal and coworkers [39] discovered that for live cells being <1% of the live/dead mixture, the quantification by viability-qPCR was not reliable.

It has been suggested that the amplification of longer target regions might increase the chance that at least one dye molecule will bind to the targeted DNA region in damaged cells and as such decrease the probability that DNA from damaged cells is amplified [40,41]. In the present study, the region amplified was 95 bp, which might be prolonged to approximately 200 bp (which is the maximal target length for qPCR).

In summary, we optimized conditions of viability-qPCR for quantification of killing of planktonic cells of four different *Gardnerella* species by means of the PM-477 endolysin. We further validated this protocol in the clinical environment of vaginal samples from women with bacterial vaginosis. We were able to document up to log2.2 (99.4% reduction) reduction of *Gardnerella* cells after treatment with PM-477, despite the complex compositions of the samples. For the samples with the highest initial loads (log7 genomes/mL), we could achieve 95.5–98.7% (97.5% average) reduction in viable cells. Given that, as deduced above, the method of viability-qPCR may be reliable only when the live cells constitute between 1 and 100% of the sample; these values indicate that treatment with PM-477 reduced live *Gardnerella* to or below our limit of detection. The high Cq values (i.e., low number of live *Gardnerella*) measured in PM-477 treated samples cannot discriminate between remaining live cells and DNA from dead cells that has not been labelled by PMA.

Viability-qPCR remains a potential method for differentiation between live and dead bacterial cells in clinical samples, including clinical samples with biofilms [9,42], but seems to require optimization specific for different bacterial species and different sample types. Although it is not an absolute quantification method, documentation of full eradication, as is possible for the culture of planktonic cells, is difficult with viability-qPCR, which has higher quantification power than FISH. We previously found the FISH method to be only semi-quantitative whereby the true degree of killing of *Gardnerella* cells was underestimated, possibly due to the staining of dead cells that still stick to the vaginal epithelial cells [20].

In the present study, the bactericidal effect of endolysin PM-477, recently clearly documented to enable full eradication of planktonic *Gardnerella* cells [20], was confirmed in vaginal samples. We established viability-qPCR, which can detect reductions in live planktonic *Gardnerella* cells of up to log4.0. In vaginal samples from women with BV, we measured strongly significant reductions of up to 99.4% (log2.2) of *Gardnerella* cells. Reductions of *Gardnerella* beyond this value were not measured. This might mean that the endolysin failed to eradicate the last cells from the vaginal biofilms. However, we deem it more likely that a higher log reduction was achieved but could not be documented as a consequence of technical limitations of the principle of viability-qPCR. At present, it is not possible to decide which is the case, because there are no valuable alternatives to confirm full eradication of (*Gardnerella*) cells from (vaginal) biofilms in clinical samples.

## Figures and Tables

**Table 1:** Different PMAxx treatment protocols of log8.19 (±0.17) *G. swidsinskii* cells incubated with PM-477 overnight in a Concept 400, Anaerobic Workstation, 75% humidity, 37 °C.

PMAxx (µM)	Number of Treatments	DMSO (%)	Temperature of Incubation (°C)	Time of Incubation (min)
0	0	0	Ice	15
25	1	0	RT	10
25	1	0	RT	15
25	1	0	Ice	15
25	1	2	Ice	15
25	1	0	37	15
25	1	0	42	15
25	2	0	Ice	15
50	1	0	Ice	15
50	2	0	Ice	15
50	3	0	Ice	15
100	1	0	RT	10
100	1	0	RT	15
100	1	0	Ice	15
100	1	2	Ice	15
100	1	0	37	15
100	1	0	42	15
100	2	0	Ice	15

Legend: RT: room temperature. On ice temperature corresponds to 0.3 °C.

**Table 2:** Primers used for qPCR.

Species/Genus	Primer Name	Primer Sequence (5′–3′)
*G. leopoldii*	GldnaG_F	GATACTGCACTGTATCGA
	GldnaG_R	CAGTATCAATACCAGCC
*G. piotii*	GpdnaG_F	AGCTGCTTACGATTATAGT
	GpdnaG_R	TTACTCATTCTAAGCTTAATAG
*G. swidsinskii*	GsdnaG_F	ATTTAGTTAGATATTTGGCAA
	GsdnaG_R	ATAGTCATATATTCCGCGC
*G. vaginalis*	GvdnaG_F	TATTATAACTAAAGCTGCTG
	GvdnaG_R	TCGCCACTATAGTCG
*Lactobacillus*	*Lactobacillus*-Fw	AGCAGTAGGGAATCTTCCA
	*Lactobacillus*-Rv	CACCGCTACACATGGAG

## Data Availability

Data is contained within the article (as Figure 1, Figure 2, Figure 3, Figure 4 and Figure 5) or Supplementary Materials. Raw data from Figure 1, Figure 2, Figure 3, Figure 4 and Figure 5 are available on request from the corresponding author.

## Supplementary Materials

The following supporting information can be downloaded at: https://www.mdpi.com/article/10.3390/antibiotics11010111/s1, Table S1: Purified DNA inactivation via PMAxx treatment examined with *G.swidsinskii*-specific qPCR. Table S2: Log genomes/mL reduction of heat killed cells compared to live cells examined with viability-qPCR (after PMAxx treatment). Table S3: CFU/mL after MES and PM-477 treatment as determined by culture. Table S4: Detection of different log genome/mL of live cells in the presence of log9/log8 dead cells using viability-qPCR.

## References

[1] Nocker A., Cheung C.Y., Camper A.K. (2006). Comparison of propidium monoazide and ethidium monoazide for differentiation of live vs. dead bacteria by selective removal of DNA from dead cells. J. Microbiol. Methods.

[2] Pan Y., Breidt F. (2007). Enumeration of viable *Listeria monocytogenes* cells by real-time PCR with propidium monoazide and ethidium monoazide in the presence of dead cells. Appl. Environ. Microbiol..

[3] Yang X., Badoni M., Gill C.O. (2011). Use of propidium monoazide and quantitative PCR for differentiation of viable *Escherichia coli* from *E. coli* killed by mild or pasteurizing heat treatments. Food Microbiol..

[4] Nkuipou-Kenfack E., Engel H., Fakih S., Nocker A. (2013). Improving efficiency of viability-PCR for selective detection of live cells. J. Microbiol. Methods.

[5] Reyneke B., Ndlovu T., Khan S., Khan W. (2017). Comparison of EMA-, PMA- and DNase qPCR for the determination of microbial cell viability. Appl. Microbiol. Biotechnol..

[6] Gardner H.L., Dukes C.D. (1954). New etiologic agent in nonspecific bacterial vaginitis. Science.

[7] Leopold S. (1953). Heretofore undescribed organism isolated from the genitourinary system. US Armed Forces Med. J..

[8] Swidsinski A., Mendling W., Loening-Baucke V., Ladhoff A., Swidsinski S., Hale L.P., Lochs H. (2005). Adherent biofilms in bacterial vaginosis. Obstet. Gynecol..

[9] Hardy L., Jespers V., Van den Bulck M., Buyze J., Mwambarangwe L., Musengamana V., Vaneechoutte M., Crucitti T. (2017). The presence of the putative *Gardnerella vaginalis* sialidase A gene in vaginal specimens is associated with bacterial vaginosis biofilm. PLoS ONE.

[10] Ahmed A., Earl J., Retchless A., Hillier S.L., Rabe L.K., Cherpes T.L., Powell E., Janto B., Eutsey R., Hiller N.L. (2012). Comparative genomic analyses of 17 clinical isolates of *Gardnerella vaginalis* provide evidence of multiple genetically isolated clades consistent with subspeciation into genovars. J. Bacteriol..

[11] Catlin B.W. (1992). *Gardnerella vaginalis*: Characteristics, clinical considerations, and controversies. Clin. Microbiol. Rev..

[12] Turovskiy Y., Sutyak Noll K., Chikindas M.L. (2011). The aetiology of bacterial vaginosis. J. Appl. Microbiol..

[13] Castro J., França A., Bradwell K.R., Serrano M.G., Jefferson K.K., Cerca N. (2017). Comparative transcriptomic analysis of *Gardnerella vaginalis* biofilms vs. planktonic cultures using RNA-seq. NPJ Biofilms Microbiomes.

[14] Paavonen J., Mangioni C., Martin M.A., Wajszczuk C.P. (2000). Vaginal clindamycin and oral metronidazole for bacterial vaginosis: A randomized trial. Obstet. Gynecol..

[15] Sobel J., Peipert J.F., McGregor J.A., Livengood C., Martin M., Robbins J., Wajszczuk J.P. (2001). Efficacy of clindamycin vaginal ovule (3-day treatment) vs. clindamycin vaginal cream (7-day treatment) in bacterial vaginosis. Infect. Dis. Obstet. Gynecol..

[16] Colli E., Landoni M., Parazzini F. (1997). Treatment of male partners and recurrence of bacterial vaginosis: A randomised trial. Genitourin. Med..

[17] Bannatyne R.M., Smith A.M. (1998). Recurrent bacterial vaginosis and metronidazole resistance in *Gardnerella vaginalis*. Sex. Transm. Infect..

[18] Eriksson K., Carlsson B., Forsum U., Larsson P.-G. (2005). A double-blind treatment study of bacterial vaginosis with normal vaginal lactobacilli after an open treatment with vaginal clindamycin ovules. Acta Derm. Venereol..

[19] Fischetti V.A. (2010). Bacteriophage endolysins: A novel anti-infective to control Gram-positive pathogens. Int. J. Med. Microbiol..

[20] Landlinger C., Tisakova L., Oberbauer V., Schwebs T., Muhammad A., Latka A., Van Simaey L., Vaneechoutte M., Guschin A., Resch G. (2021). Engineered phage endolysin eliminates *Gardnerella* biofilm without damaging beneficial bacteria in bacterial vaginosis ex vivo. Pathogens.

[21] Schmelcher M., Donovan D.M., Loessner M.J. (2012). Bacteriophage endolysins as novel antimicrobials. Future Microbiol..

[22] Drulis-Kawa Z., Majkowska-Skrobek G., Maciejewska B., Delattre A.-S., Lavigne R. (2012). Learning from bacteriophages-Advantages and limitations of phage and phage-encoded protein applications. Curr. Protein Pept. Sci..

[23] Schuch R., Pelzek A.J., Nelson D.C., Fischetti V.A. (2019). The PlyB endolysin of bacteriophage vB_BanS_Bcp1 exhibits broad-spectrum bactericidal activity against *Bacillus cereus sensu lato* isolates. Appl. Environ. Microbiol..

[24] Vaara M. (1992). Agents that increase the permeability of the outer membrane. Microbiol. Rev..

[25] Vaneechoutte M., Guschin A., Van Simaey L., Gansemans Y., Van Nieuwerburgh F., Cools P. (2019). Emended description of *Gardnerella vaginalis* and description of *Gardnerella leopoldii* sp. nov., *Gardnerella piotii* sp. nov. and *Gardnerella swidsinskii* sp. nov., with delineation of 13 genomic species within the genus *Gardnerella*. Int. J. Syst. Evol. Microbiol..

[26] Vaneechoutte M. (2017). The human vaginal microbial community. Res. Microbiol..

[27] Nocker A., Mazza A., Masson L., Camper A.K., Brousseau R. (2009). Selective detection of live bacteria combining propidium monoazide sample treatment with microarray technology. J. Microbiol. Methods.

[28] Nocker A., Camper A.K. (2009). Novel approaches toward preferential detection of viable cells using nucleic acid amplification techniques. FEMS Microbiol. Lett..

[29] Kobayashi H., Oethinger M., Tuohy M.J., Hall G.S., Bauer T.W. (2009). Improving clinical significance of PCR: Use of propidium monoazide to distinguish viable from dead *Staphylococcus aureus* and *Staphylococcus epidermidis*. J. Orthopaed. Res..

[30] Nocker A., Sossa-Fernandez P., Burr M.D., Camper A.K. (2007). Use of propidium monoazide for live/dead distinction in microbial ecology. Appl. Environ. Microbiol..

[31] Kralik P., Nocker A., Pavlik I. (2010). *Mycobacterium avium* subsp. *paratuberculosis* viability determination using F57 quantitative PCR in combination with propidium monoazide treatment. Int. J. Food Microbiol..

[32] Vaneechoutte M. (2017). *Lactobacillus iners*, the unusual suspect. Res. Microbiol..

[33] Emerson J.B., Adams R.I., Betancourt Román C.M., Brooks B., Coil D.A., Dahlhausen K., Ganz H.H., Hartmann E.M., Hsu T., Justice N.B. (2017). Schrödinger’s microbes: Tools for distinguishing the living from the dead in microbial ecosystems. Microbiome.

[34] Pisz J.M., Lawrence J.R., Schafer A.N., Siciliano S.D. (2007). Differentiation of genes extracted from non-viable versus viable micro-organisms in environmental samples using ethidium monoazide bromide. J. Microbiol. Methods.

[35] Steinberger R.E., Holden P.A. (2005). Extracellular DNA in single- and multiple-species unsaturated biofilms. Appl. Environ. Microbiol..

[36] Fittipaldi M., Codony F., Adrados B., Camper A.K., Morató J. (2011). Viable real-time PCR in environmental samples: Can all data be interpreted directly?. Microb. Ecol..

[37] Liang Z., Keeley A. (2012). Comparison of propidium monoazide-quantitative PCR and reverse transcription quantitative PCR for viability detection of fresh *Cryptosporidium* oocysts following disinfection and after long-term storage in water samples. Water Res..

[38] Wang L., Li Y., Musthapha A. (2009). Detection of viable *Escherichia coli* O157:H7 by ethidium monoazide real time PCR. J. Appl. Microbiol..

[39] Løvdal T., Befring Hovda M., Björkblom B., Møller S.G. (2011). Propidium monoazide combined with real-time quantitative PCR underestimates heat-killed *Listeria innocua*. J. Microbiol. Methods.

[40] Banihashemi A., Van Dyke M.I., Huck P.M. (2012). Long-amplicon propidium monoazide-PCR enumeration assay to detect viable *Campylobacter* and *Salmonella*. J. Appl. Microbiol..

[41] Contreras P.J., Urrutia H., Sossa K., Nocker A. (2011). Effect of PCR amplicon length on suppressing signals from membrane-compromised cells by propidium monoazide treatment. J. Microbiol. Methods.

[42] Deschaght P., Schelstraete P., Van Simaey L., Vanderkercken M., Raman A., Mahieu L., Van Daele S., De Baets F., Vaneechoutte M. (2013). Is the improvement of CF patients, hospitalized for pulmonary exacerbation, correlated to a decrease in bacterial load?. PLoS ONE.

